# The fate of nitrogen in the Zarin-Gol River receiving trout farm effluent

**DOI:** 10.1038/s41598-023-49243-6

**Published:** 2023-12-08

**Authors:** Altin Ghojoghi, Rasoul Ghorbani, Rahman Patimar, Abdolrassoul Salmanmahiny, Rahmat Naddafi, Abdolazim Fazel, Timothy D. Jardine

**Affiliations:** 1 Department of Fisheries, Gorgan University of Agricultural Sciences and Natural Resources, Gorgan, Iran; 2 Department of Fisheries, Gonbad Kavous University, Gonbad Kavous, Iran; 3 Department of Environment, Gorgan University of Agricultural Sciences and Natural Resources, Gorgan, Iran; 4 Department of Aquatic Resources, Swedish University of Agricultural Sciences, Uppsala, Sweden; 5 Inland Waters Aquatic Resources Research Center, Iranian Fisheries Science Research Institute, Gorgan, Iran; 6 School of Environment and Sustainability, University of Saskatchewan, Saskatoon, Canada

**Keywords:** Freshwater ecology, Stable isotope analysis

## Abstract

This study investigated the Zarrin-Gol River ecosystem in Iran to trace organic matter in the food web and evaluate the impact of aquaculture farm effluent using stable isotopes of nitrogen (δ^15^N) and carbon (δ^13^C). Using a previously-developed model (Islam 2005), we estimated that a trout farm in the vicinity released 1.4 tons of nitrogen into the river. This was comparable to an estimated total nutrient load of 2.1 tons of nitrogen for the six-month fish-rearing period based on a web-based constituent load estimator (LOADEST). A model estimate of river nitrogen concentration at the time of minimum river discharge (100 L/s) was 2.74 mg/L. Despite relatively high nitrogen loading from the farm, isotope data showed typical food web structure. Several biological groups had elevated δ^13^C or δ^15^N values, but there was limited evidence for the entry of organic matter from the trout farm into the food web, with sites above and below trout farms having inconsistent patterns in ^15^N enrichment. By coupling nitrogen load modeling with stable isotope analysis we showed that stable isotopes might not be effective tracers of organic matter into food webs, depending on surrounding land use and other point sources of nutrients. The Zarrin-Gol River ecosystem, like other basins with high human population density, remains vulnerable to eutrophication in part due to trout farm effluent.

## Introduction

Freshwater ecosystems are under duress with changes to water quality and quantity from the combined effects of land use change, industrial pollution, and climate change^1^. These changes can affect biodiversity and productivity of freshwater ecosystems^2^, ultimately to the detriment of human well-being through alteration of ecosystem services^3^. Cultural eutrophication is one such process attributed to human activity, driven by point source and non-point source releases of limiting nutrients^4^. While agricultural runoff and discharges from municipal and industrial wastewater are well known sources of excess nutrients^5,6^, one relatively understudied potential point source of nutrients to freshwaters is the production of fish in aquaculture operations^7^.

In general, optimizing feeding efficiency and minimizing nutrient loading into receiving waters are crucial in any aquaculture activity^8^. Nitrogen (N) and phosphorus (P) produced by fish metabolism are the primary sources of dissolved N and P wastes in intensive aquaculture operations. Elevated production of these two elements in the effluent of aquaculture systems leads to eutrophication and, consequently, ecological alterations in downstream aquatic ecosystems^9^. The amount of feeding residues in aquaculture is influenced by factors such as breeding extent, fish species, rearing methods, and food characteristics^10,11^. The expansion in fish farming activities has significantly increased the quantity of particulate food residues and wastes in the environment. This increase is in tandem with other human activities that significantly modify the N cycle in terrestrial and aquatic ecosystems, amplifying the N burden in rivers. Less than 30% of the human-derived N released in freshwaters reaches the oceans through runoff and rivers, with over 70% of N inputs being retained or recycled within watersheds. Although there is evidence that this loaded N undergoes processing in watersheds, it is difficult to determine its origins, especially when multiple sources are present^12^.

The unique hydrological characteristics of rivers make them vulnerable to various pollutants, both directly and indirectly. Therefore, the assessment and management of nutrients in river systems have been crucial issues in water resource management in recent decades^13^. Organic matter and nutrients are assimilated by primary producers or directly consumed by consumers, thus entering food webs.

Understanding the nutritional structure and energy flow can provide valuable insights into ecosystem management and conservation^11^. Stable isotope analysis (SIA) is a powerful tool that can be used to trace the uptake of nutrients (minerals) by primary producers and secondary consumers in both terrestrial and marine ecosystems^14–16^. As the isotope ratios are determined by the type of diet, they can be used to investigate the sources of nutrients for aquatic organisms as well as their nutritional status^17^. However, the quantity and quality of food may vary in different locations and times, resulting in spatio-temporal fluctuations in the stable isotope values (δ^13^C and δ^15^N) of consumers. Isotopic techniques have been used in several studies to identify sources of N and to describe changes in N in terrestrial and aquatic ecosystems^18^.

Changes in the natural abundance of ^15^N in sediments, suspended particulate matter, macro algae, rooted macrophytes, bivalves, gastropods, invertebrates, zooplankton, and fish can be attributed to human N inputs from wastewater and agricultural activities^19,20^. Lake et al. (2001)^21^ traced N loading with δ^15^N, demonstrating that urban development and increased human wastewater led to an increase in δ^15^N in biota from 17 small freshwater systems. Nitrogen isotopes in fish larvae have been successfully applied as a marker in identifying the internal sources of nutrients in estuaries and wetlands, with ammonium concentrations having a significant relationship with isotopic values^22^. Stable N and carbon (C) isotopes have also become widely used in studying food webs, including predation patterns, feeding structure, and trophic exchange in various ecosystems^23^. In trophic interactions, the consumer tends to be isotopically heavier than the food source, a phenomenon known as the trophic enrichment factor (Δ^13^C and Δ^15^N for C and N, respectively)^24^. This effect is more pronounced in N, with a δ^15^N increase of 3–4 ‰ at each trophic level. The ratio of C isotopes (δ^13^C) varies only slightly with the trophic transfer, usually less than 1‰ from prey to predator^25^.

Numerous studies have demonstrated the adverse impacts of aquaculture on nutrient status of marine waters. Islam (2005)^26^ employed a model to estimate that 132.5 kg of N is discharged into the environment per ton of fish produced through cage culture. The amounts of N, P, and C released into marine cages located in Mazandaran province by farming rainbow trout (*Oncorhynchus mykiss*) were reported by Yazdani et al. (2020)^27^, for every ton of fish produced, 74 kg of N and 14 kg of P enter the environment. An evaluation of the environmental impact of farming rainbow trout pens in the southern Caspian Sea found that the total amounts of N and P released were 0.76 and 0.164 tons, respectively^28^.

While less common, there have been some studies on aquaculture discharges in rivers, and results have been mixed. Varol and Balci (2020)^29^ examined the characteristics of the effluent of rainbow trout farms in Turkey and its effect on water quality, finding a significant increase in total P concentrations. Evaluation of the impact of traditional rainbow trout farming on receiving water quality in Ireland conducted by Tahar et al*.* (2018)^30^ showed no significant effect on the river water quality. Oenema et al. (2005)^31^ revealed that the rate of nitrate infiltration into groundwater as well as the discharge of N and P to surface water are influenced by hydrological status, land use, and soil type. In the Chalous River of northern Iran, Mirbagheri et al. (2011)^32^ employed the river self-purification model and estimated N change. They found that the river's self-purification rate was low, and there was a significant increase in the amount of organic N where wastewater enters the river. Also, the effect of aquaculture waste in Lake Poyang on the stable isotope composition of C and N from organic sediment, fish feed, and fish feces was examined by Wang et al. (2020)^33^. Results showed the impact of aquaculture waste on the organic content of sediment. Yet studies that couple nutrient modeling and isotope analysis are rare.

Intensive farming and sewage discharge have polluted terrestrial and aquatic ecosystems with high levels of nitrogen and phosphorus^34^. N and P concentrations are usually very high in waters draining cultivated landscapes, due to the application of fertilizers and soil management practices^35^. Predicting how ecological communities will respond to human and natural changes to the environment requires a combination of models and empirical data that explore the response to multiple stressors at the species, community and ecosystem scales. Due to the presence of agricultural activities and fish farms in the Zarin-Gol River^36^, it is necessary to investigate the possible impacts of agricultural and aquaculture activities in sensitive ecosystems such as dryland rivers of Iran. To that end, we combined stable isotope analysis of riverine biota at various locations along a river receiving aquaculture effluent with multiple nutrient loading models to determine the source and fate of nutrients. We used this approach to track nitrogen pollutant sources in the dominant biotic and abiotic sources in the Zarin-Gol River.

## Methods

### Study area and sampling

**Figure 1:**
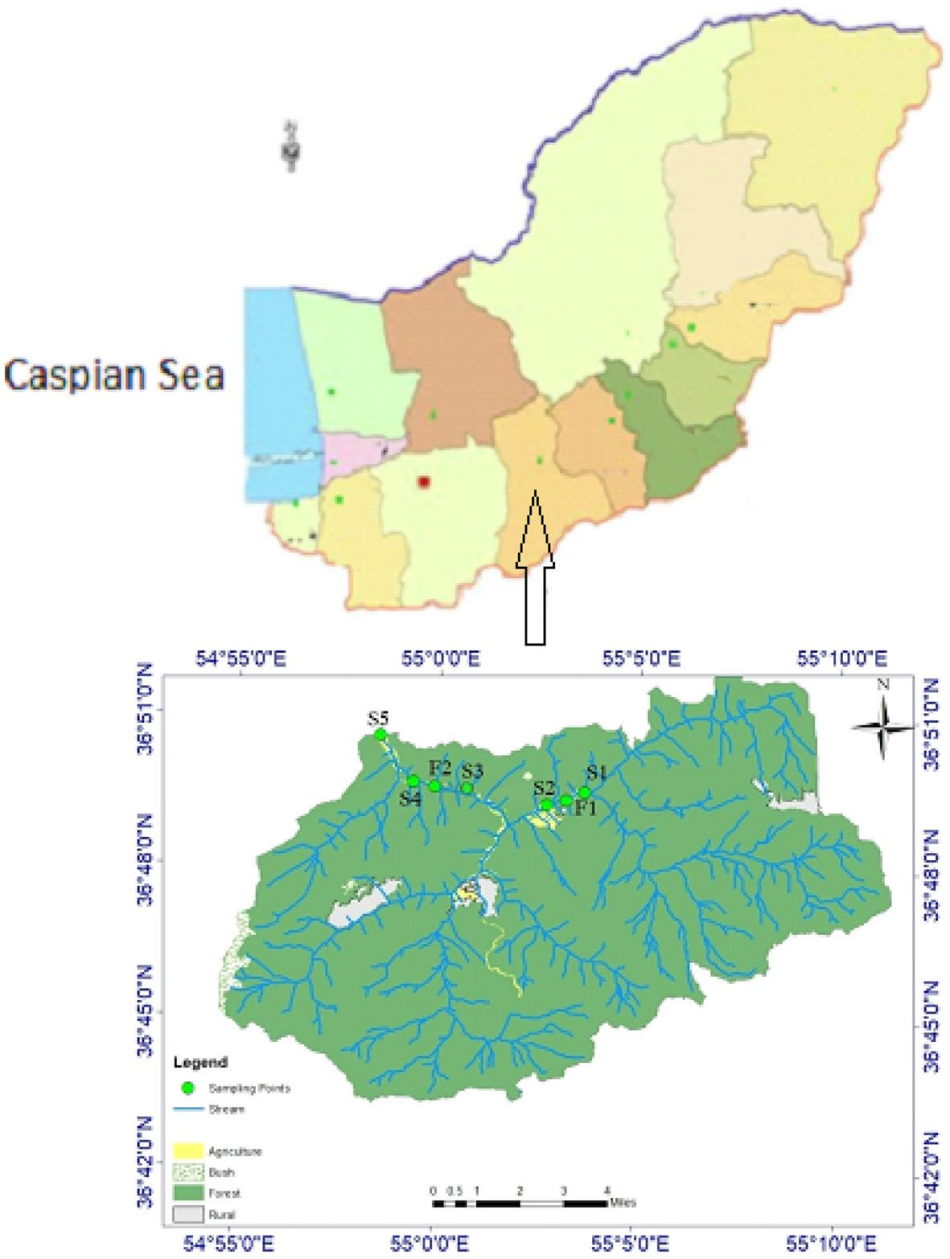
Map of the study area illustrating fish farms (F1 and F2) and sampling points (S1–S5) in the Zarin-Gol River, northern Iran. The process of crafting the map began with the initial steps of determining the watershed using Digital Elevation Model data from STRM^37^ and the GRASS tool in QGIS, with the data source accessible at https://portal.opentopography.org/datasetMetadata. Subsequently, a comprehensive land use analysis unfolded through the digitization of Google Earth images, extraction of land use polygons, and their conversion into shapefiles using QGIS version 3.30.2; additional details about QGIS can be found at https://www.qgis.org/en/site. The integration of geographic coordinates for farms into QGIS marked a pivotal phase, concluding with the meticulous crafting of the legend and the establishment of the geographic coordinate system within the QGIS environment.

The study was conducted from spring 2019 to winter 2020 in the Zarrin-Gol River, which is a small part of the Gorgan-Rud basin located in the southern Caspian Sea (Fig. 1). After conducting a preliminary field visit to the river and taking into account the various land uses along the river and the introduction of fish farm effluents into the river, five sampling sites were selected. These sites include Site 1, which is representative of an area upstream of the farms (just before the first farm) (S1), Site 2, which is located 200 m after the first fish farm effluent entry into the river (S2), Site 3, which is just before the second fish farm effluent entry into the river (S3), Site 4, which is 200 m after the second fish farm effluent entry into the river (S4), and Site 5, which is located 1000 m after the second fish farm effluent entry into the river (S5). The sites F1 and F2 are fish farms (as shown in Fig. 1). Additionally, the predominant land uses surrounding the river, such as agriculture, forestry, and rainbow trout farming, were determined and coded on the maps.

Data pertaining to the second fish farm's production was collected during a 6-month fish rearing period. This data encompassed the total rearing capacity of 30 tons, comprising 10 raceways, each containing 35,000 fish per pond with an initial weight of 60 g per fish (group A), and 5 raceways, each containing 12,000 fish per pond with an initial weight of 50 g per fish (group B). Moreover, various environmental parameters were measured through the use of spectrophotometry in the laboratory. These parameters include nitrate (mg/L) and nitrite (mg/L), ammonia/ammonium (mg/L), dissolved phosphate (mg/L), and total P (mg/L).

In line with the objective of this study, samples (19 total) were collected from three fish species including: eight samples of *Capoeta razii*, two samples of *Neogobius pallasi*, and nine samples of *Paracobitis hircanica*. In addition, periphyton (1 sample), and aquatic insect larvae (5 samples) were collected from different stations in the spring.

A spatula was used to gather periphyton from rock surfaces, and these samples were then placed on ice and transferred to the laboratory. Mud was removed from the pooled sample using distilled water, following which the sample was subjected to freeze drying for 48 h. Finally, the sample was pulverized. For collecting benthos, a Surber sampler (specifically designed for running water) was employed. The samples were then washed using a sieve with a 0.5 mm mesh size and transported to the laboratory while being kept on ice. The samples were identified at the family level in the laboratory and subsequently kept in the freeze dryer for 48 h. Whole samples were then pulverized and stored in microtubes.

Sampling of juvenile fish (size 4–6 cm) was conducted using an electro-shocker with a voltage of 200–300 V. 3–5 samples were collected from each site, placed on ice, and then transported to the laboratory, where they were stored in a −80 °C freezer. Following a 24-h period, the samples underwent a thorough cleansing with deionized water before being freeze-dried for 48 h. They were then pulverized and stored in microtubes, each labeled appropriately, and sent to the laboratory at the Swedish University of Agricultural Sciences in Uppsala for isotopic analysis through the use of EA-IRMS.

Stable isotope values (δ^13^C and δ^15^N) were analyzed for each sample using a stable isotope ratio mass spectrometer, employing the formula δX = [(R sample/R standard) – 1] × 1000, where X represents the value of ^13^C or ^15^N and R represents the ratio ^12^C:^13^C or ^14^N:^15^N. The standard deviation of a secondary standard analyzed in parallel with the samples was found to be less than 0.15‰ and 0.16‰ for δ^13^C and δ^15^N, respectively.

### Data analysis and modeling

**Figure 2:**
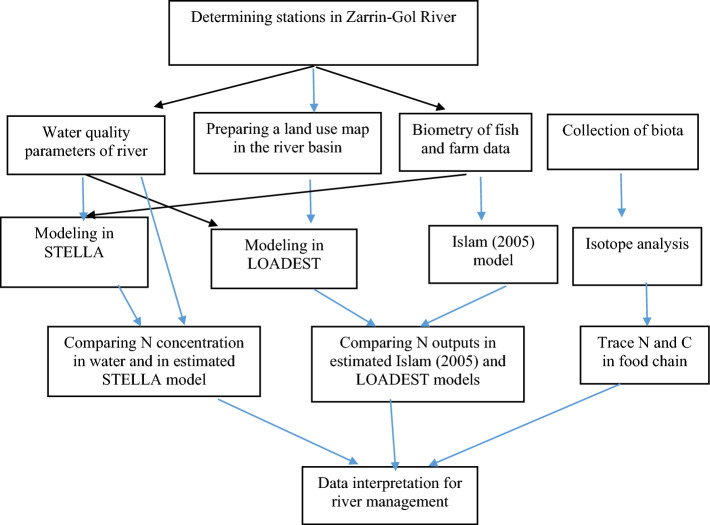
A schematic of data collection and modeling processes.

Figure 2 shows the schematic of how the data was collected, and how the modeling processes were performed. Water quality data were first analyzed for spatial variation using one-way Analysis of Variance (ANOVA) followed by a LSD test for comparing means, with alpha set at 0.05 and using SPSS16. Utilizing production data from the second fish farm, we determined the nutrient loads entering the river from the farm. We accomplished this by subtracting the biomass of N in the fish from the total N supplied by food during the previous half-year of rearing, taking into account expected nutrient ratios (6.5% for N in required feed and 3% for N in harvested fish). Subsequently, we employed Islam’s (2005) model to calculate the anticipated N load while also accounting for feed loss and varying food conversion rates, according to Golaghaei Farabi et al. (2021)^38^.

A nitrogen dynamic mathematical model was created using the advanced STELLA software version 9.0.1. A conceptual model was set based on Odum et al*.* (1983)^39^ who showed the process of nitrogen consumption and production in the breeding unit during the period of entry, transformation and exit from the water column for aquaculture systems. The model outlines the flow of N in the river, taking into account the interaction between the fish farm and the environment. STELLA is software for simulation and modeling that allows the parameterization of stocks and flows. It visualizes the content dynamically to understand and illustrate system complexity. By connecting the input and output data, relationships between variables can be examined^40,41^. This model was employed to estimate the pollutant load of the Zarrin-Gol River using total N before and after the fish farm. Also, by using the total N and daily water discharge of the Zarrin-Gol River, the N load in the basin and the fish farm were estimated with the assistance of web-based load calculation software (LOADEST, https://water.usgs.gov/software/loadest/, Park et al. 2015)^42^. This software is used to calculate total maximum daily loads, the permissible load of different rivers that depends on various temporal and spatial factors related to the type and intensity of the incoming residual material and the environmental conditions within the river. The TMDL process is used to determine self-treatment capacity and load under defined scenarios, and involves estimating the maximum amount of pollutants a water body can absorb from point and nonpoint sources and establishing water quality standards.

Although direct isotope data from the fish farm was not available, we estimated that excess feed and waste from rainbow trout culture operations have δ^15^N ~ 7‰ and δ^13^C ~ -22‰, based on previous research conducted by Hurd et al. (2008)^43^ and Kullman et al. (2009)^44^. Consequently, we predicted that biota consuming N and C originating from trout farms would exhibit elevated isotope values.

### Ethics statement

All experimental protocols were approved by Gorgan University of Agricultural Sciences and Natural Resources, Gorgan, Iran (No. 9621074180). The methods carried out in accordance with relevant guidelines and regulations. Moreover, all the methods of the present study and reporting herein follows the recommendations in the ARRIVE guidelines.

## Results

### Water quality and estimated released nitrogen (Islam 2005)

Dissolved oxygen (DO) values were reported in the range of 8–11.5 mg/L, which indicates good water quality. The lowest amount of DO was observed in summer and the highest in winter, but there were no differences among stations. *E. coli* and phosphate values were significantly different among stations. The highest values were at the station 200-m after the second fish farm (S4), and the lowest values were at the first station (before the first fish farm). There were no significant differences in pH, temperature, turbidity, total dissolved solids, electrical conductivity, salinity and nitrate in the different stations. Nitrate concentrations in the 5 stations were relatively high between 0.56 and 1.83 mg/l (Table 1).

**Table 1:** Mean physicochemical parameters measured in different stations of Zarin-Gol River in 2020.

Parameter	S1	S2	S3	S4	S5
DO	0.87 ± 9.66	1.29 ± 9.23	1.29 ± 9.16	0.17 ± 8.57	0.75 ± 8.94
pH	0.3 ± 8.47	048 ± 8.62	0.19 ± 8.75	1.49 ± 9.07	0.12 ± 8.58
*E. coli*	0.09^d^ ± 0.925	0.09^b^ ± 2.27	0.12^b^ ± 2.15	0.16^a^ ± 2.78	0.08^c^ ± 1.8
Temperature (°C)	4.13 ± 13.95	3.75 ± 13.87	5.9 ± 17.25	7.09 ± 17.7	4.14 ± 19.32
Turbidity (NTU)	25.3 ± 35.3	85.18 ± 90.47	192.38 ± 169.35	79.39 ± 92.12	93.90 ± 104.35
PO4(mg/l)	0.16^b^ ± 0.17	0.21^ab^ ± 0.35	21^ab^ ± 0.30	0.4^a^ ± 0.58	0.12^ab^ ± 0.27
NO3(mg/l)	1.18 ± 1.4	1.06 ± 1.7	0.74 ± 1.5	1.23 ± 1.83	0.43 ± 0.56
TDS(mg/l)	453.96 ± 861	229.45 ± 358.75	185.26 ± 534	208.59 ± 579.5	91.92 ± 496
EC(µ mho/cm)	122.4 ± 12.04	71.72 ± 65.5	78.09 ± 80.69	78.09 ± 85.56	62.27 ± 75.36
Salinity (g/l)	0.49 ± 0.47	0.18 ± 0.28	0.27 ± 0.35	0.28 ± 0.35	0.22 ± 0.32

Different letters indicate significant differences among stations.

Based on the Islam (2005) model and assuming the absence of natural food in the river and the nutrient dynamics in the fish farm system (Table 2), the overall influx of N into the river from the 29-ton production fish farm was approximated to be 1.4 tons.

**Table 2:** Estimated values of released nitrogen from fish farms into the ZarrinGol River (numbers on the left within cells: group A of raceways; numbers on the right within cells: group B of raceways).

Month/factors	January 2020	February 2020	March 2020	April 2020	May 2020	June 2020
Number of stocked fish	12,000 + 35,000	12,000 + 35,000	11,400 + 33,250	10,830 + 31,587	10,288 + 30,008	9774 + 28,508
Survival rate (%)	100	100	95	95	100	100
Mean weight of fish (kg)	0.05 + 0.06	0.08 + 0.09	0.15 + 0.22	0.25 + 0.34	0.4 + 0.55	0.65 + 0.8
Total biomass (ton)	0.6 + 2.1	0.96 + 3.15	1.71 + 7.31	2.71 + 10.74	4.12 + 16.5	6.35 + 22.81
Consumed food (FCR = 1.2)	0.72 + 2.52	1.15 + 3.78	2.05 + 8.78	3.25 + 12.89	4.94 + 19.81	7.62 + 27.37
Total food nitrogen (tons)	0.047 + 0.164	0.075 + 0.246	0.133 + 0.571	0.211 + 0.838	0.321 + 1.29	0.496 + 1.78
Unconsumed food (tons) = 10%	0.072 + 0.252	0.115 + 0.378	0.205 + 0.878	0.325 + 1.289	0.494 + 1.98	0.762 + 2.74
Unconsumed nitrogen (tons)	0.005 + 0.016	0.007 + 0.025	0.013 + 0.057	0.021 + 0.084	0.032 + 0.129	0.05 + 0.178
Consumed food by fish (90%)	0.65 + 2.27	1.04 + 3.4	1.84 + 7.9	2.92 + 11.6	4.44 + 17.8	6.86 + 24.6
Total nitrogen of consumed food (tons)	0.042 + 0.147	0.067 + 0.22	0.12 + 0.51	0.19 + 0.75	0.3 + 1.16	0.49 + 1.6
Nitrogen of fish meat (tons)	0.018 + 0.063	0.029 + 0.095	0.051 + 0.219	0.081 + 0.322	0.12 + 0.5	0.19 + 0.68
Excreted nitrogen in feces and urine (tons)	0.024 + 0.08	0.038 + 0.127	0.069 + 0.294	0.109 + 0.432	0.165 + 0.663	0.255 + 0.917
Total nitrogen released into the environment (tons)	0.029 + 0.1	0.046 + 0.15	0.082 + 0.35	0.13 + 0.52	0.2 + 0.79	0.3 + 1.09
Total nitrogen released from fish farm to the environment (tons)	0.13	0.2	0.43	0.65	0.99	1.4

### Estimated released nitrogen (LOADEST)

Since the river receives effluents from both aquaculture and agriculture activities in different parts of the river, the first inputs from the whole area of the river basin were calculated with QGIS3.30.2. Considering the area of the basin (Table 3) and discharge of the river, the total N loading of the river was estimated to be 236 tons per year, which includes the total basin effect (basin and first farm). The estimated total annual load of N into the river is 4.2 tons per year from the second fish farm (Table 3).

**Table 3:** Estimated released nitrogen from fish farms into Zarrin-Gol River.

Sampling Point	Area (km^2^)	Estimated total annual load (tons/year)
Before the fish farm	232.6	236
After the fish farm	290.5	240.2
Estimated effect of fish farm	57.9	4.2

### River N concentrations (STELLA)

**Figure 3:**
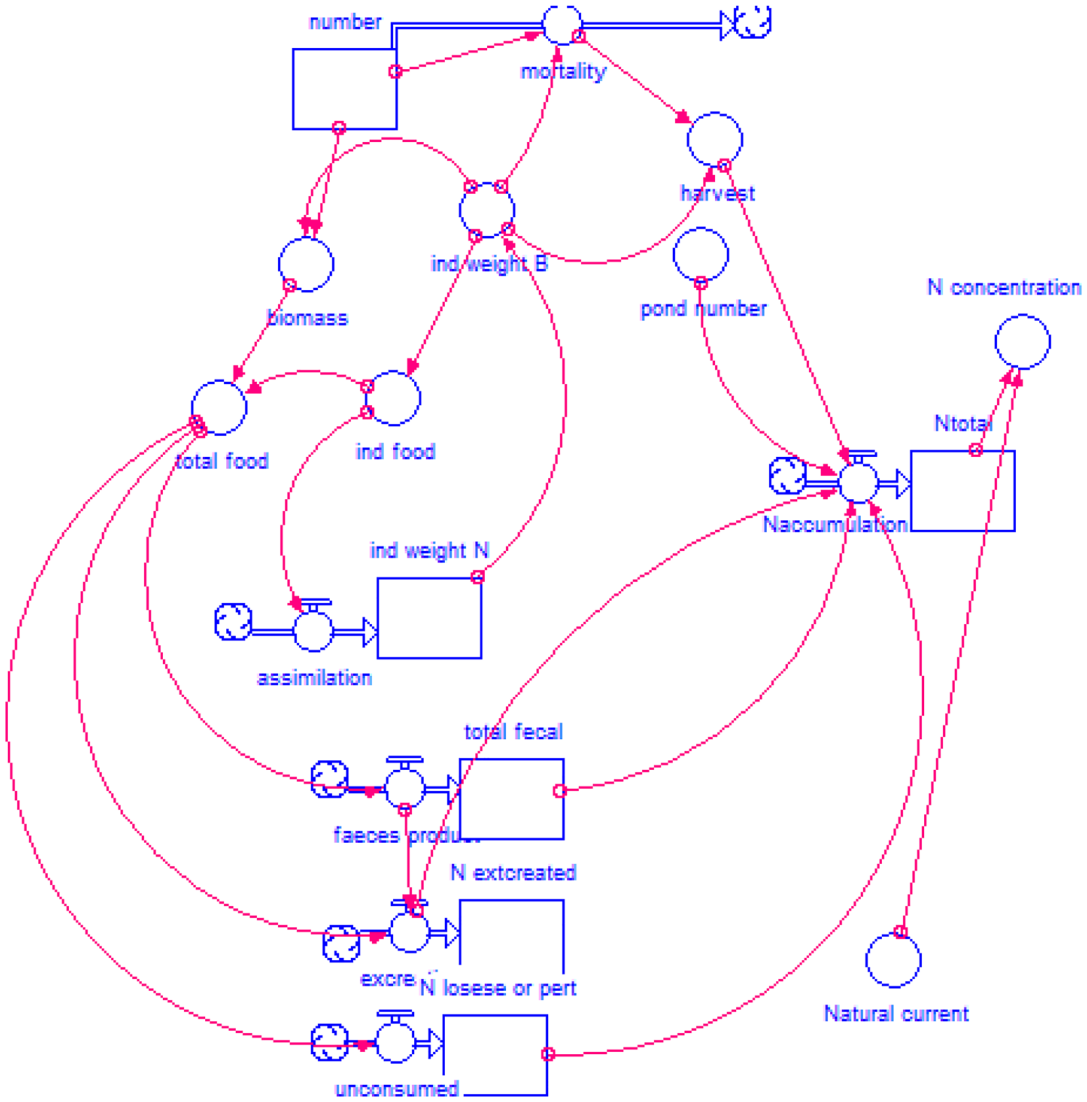
Dynamic modeling of predicted nitrogen production by the trout farm.

**Figure 4:**
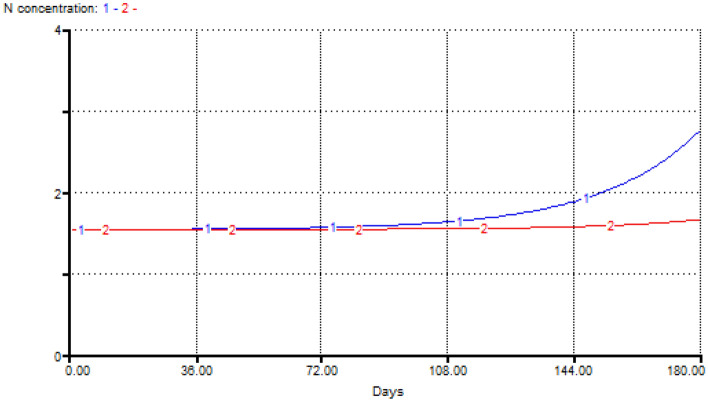
Prediction of total nitrogen production from rainbow trout farming with a density of 47,000 ind./farm (1—blue: 100 L/s—current density; 2—red: 1000 L/s—current density).

Considering the flow rate of the river and the density of fish in the farm, total N concentrations were predicted using a dynamic model in STELLA (Fig. 3; Appendix 1). As a result, at flow rates ranging from 100 to 1000 L/s (min–max of discharge of the ZarrinGol River), the concentration of N in the river will be equal to 1.65–2.74 mg/L at current production densities (Fig. 4), consistent with measured nitrate values (Table 2).

### Stable isotopes

**Figure 5:**
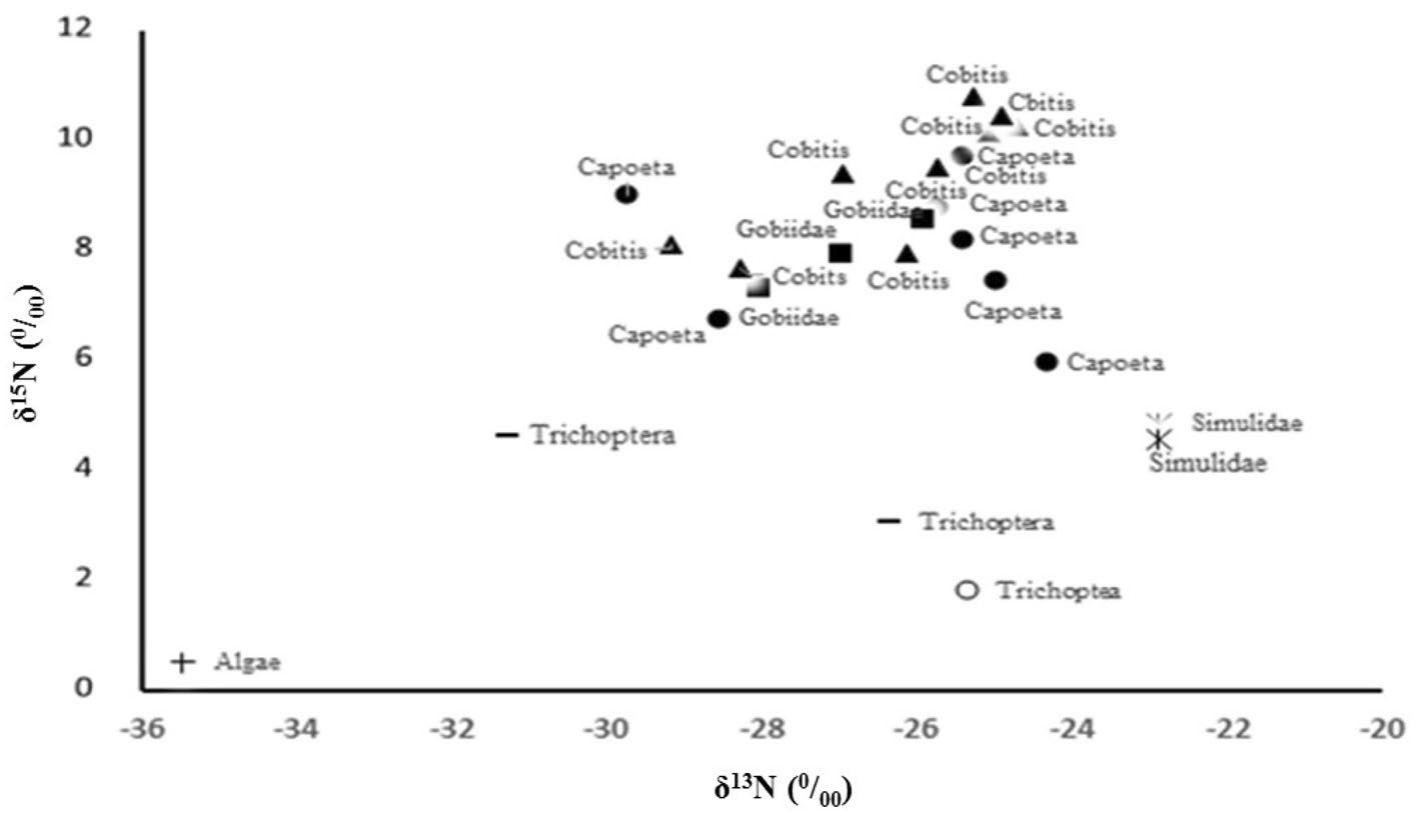
Isotope ratios of nitrogen (δ^15^N) vs. carbon (δ^13^C) in the dominant aquatic organisms in Zarrin-Gol River (all sites combined); Capoeta: *Capoeta razii*, Cobitis: *Paracobitis hircanica*, Gobiidae: *Neogobius pallasi.*

Stable isotopes of N and C in river organisms showed predictable patterns (Fig. 5). The highest values were observed in the loach (*Paracobitis hircanica*), the lowest values in the monkey goby (*Neogobius pallasi*), and intermediate values in the cyprinid (*Capoeta razii*). The fish species had higher values than the aquatic insect larvae. All benthic groups (with the exception of Trichoptera) had higher δ^15^N values than algae. δ^13^C values in Trichoptera were close to those in fish, while Simulidae had a higher value than fish and Trichoptera. The algae had the lowest δ^13^C value among the studied organisms. From algae to aquatic insect larvae, δ^15^N increased between 2.5 and 4.5‰ and from primary consumers to fish, it increased between 3.5 and 4.5‰.

**Figure 6:**
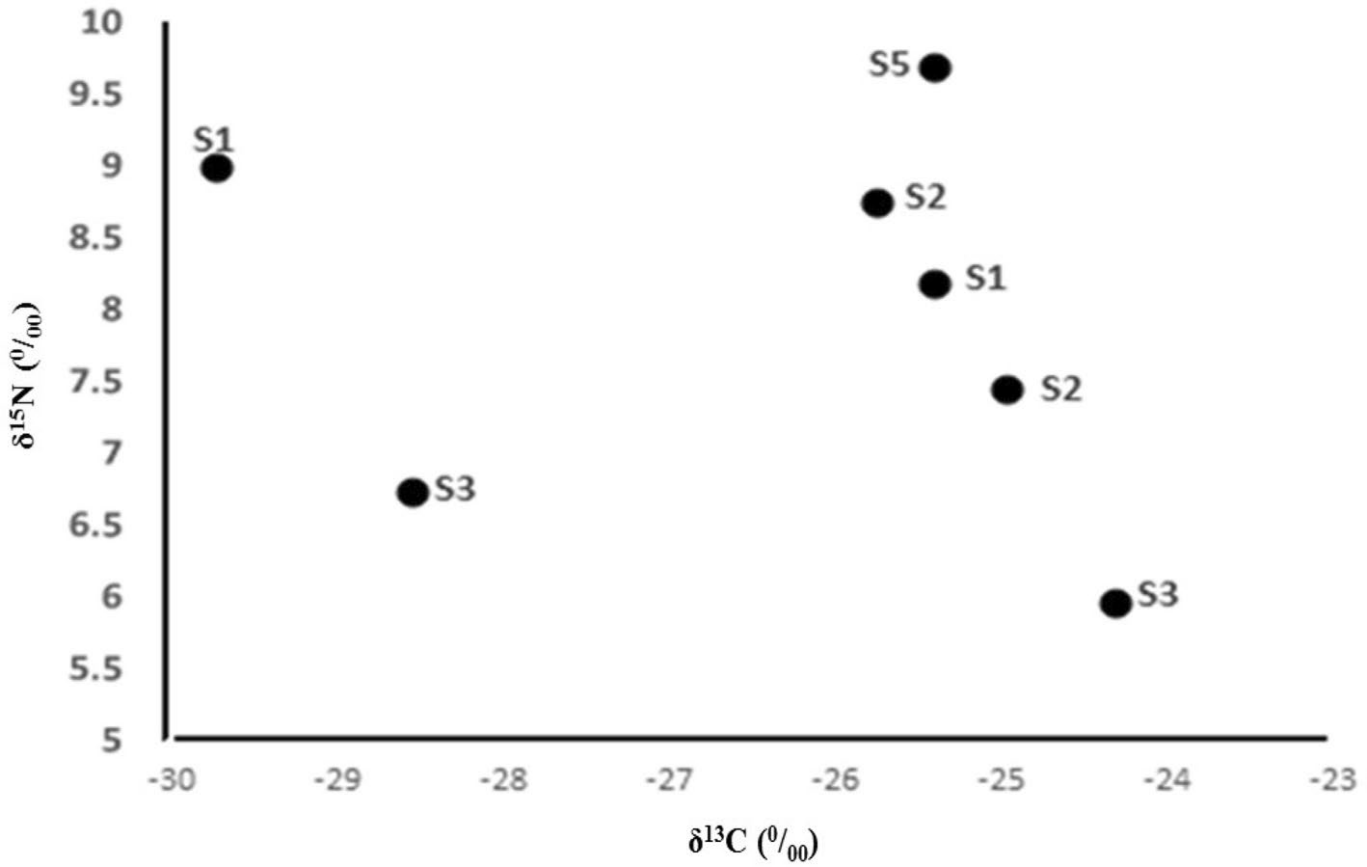
Isotope ratios of nitrogen (δ^15^N) vs. carbon (δ^13^C) in *C. razii* in different stations.

In a species collected at multiple locations (*C. razii*), we observed that the specimens from immediately below one of the fish farms (S2) had intermediate δ^15^N values that were comparable to the most upstream site above the fish farms (S1), and were lower than the most downstream site (S5) (Fig. 6). The site in the middle of the study area (S3) had the lowest δ^15^N values. δ^13^C values had limited differences between these areas (Fig. 6), with as much variation within sites as between sites and no evidence of ^13^C enrichment at the sites below fish farms.

**Figure 7:**
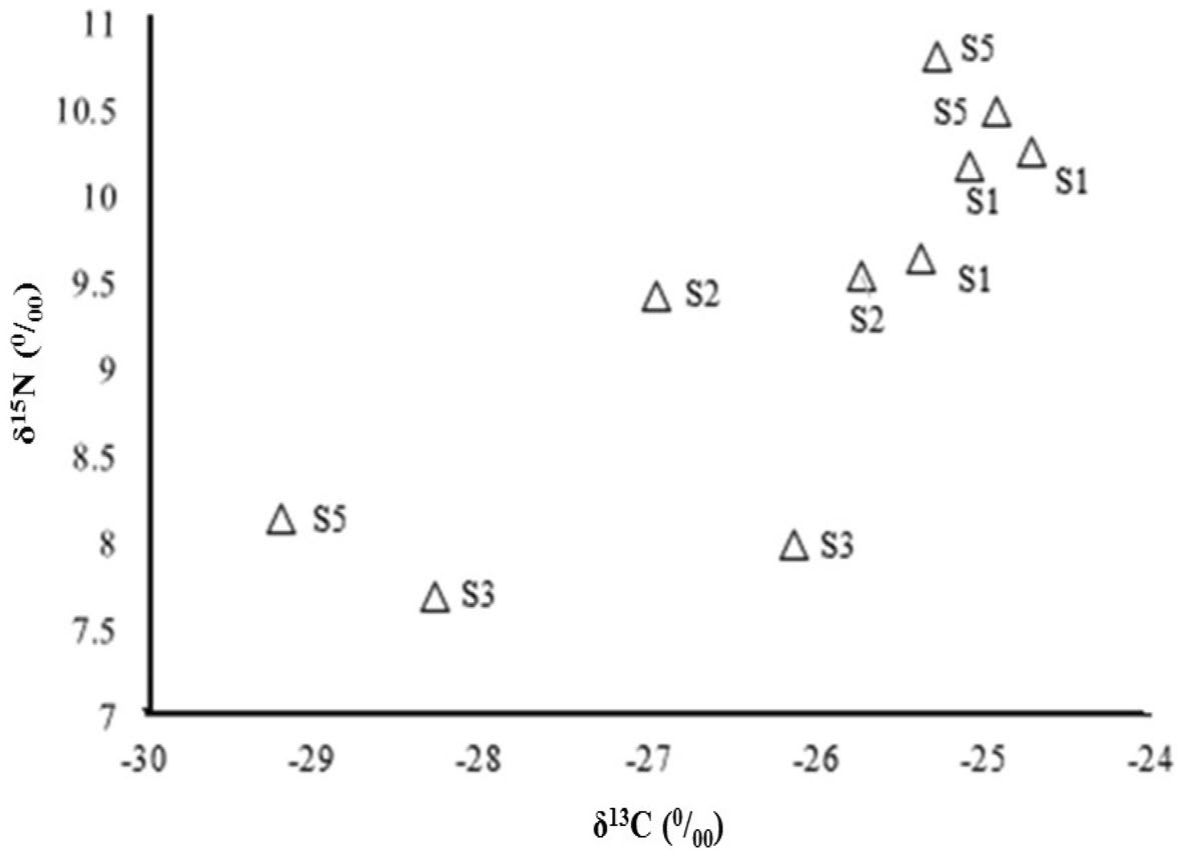
Isotope ratios of nitrogen (δ^15^N) vs. carbon (δ^13^C) in *P. hircanica* in different stations.

In *P. hircanica*, patterns were similar to those of *C. razii*, with similar, intermediate δ^15^N values at S1 and S2, despite the presence of the fish farm between these two stations. The most downstream site had the highest δ^15^N values (Fig. 7), and δ^13^C values showed no evidence for ^13^C enrichment below fish farms.

## Discussion

In the present study, we combined nutrient model outputs with values of isotopic N (δ^15^N) and C (δ^13^C) in different biological communities to examine the dynamics of a river food web. Despite the high expected N loading from trout farms, there was limited evidence for a trout waste signal in the food web. We attribute this to the fact that in the areas before the fish farm, agricultural operations, especially rice cultivation (as a thin band on both sides of the river), are the main land uses in the riverside. Also, agricultural discharges, including nitrate and phosphate fertilizers released into the river, and denitrification that increases the δ^15^N of N available for uptake by primary producers, contribute to this phenomenon^19,45^. In addition, fish farm effluents enter the river, intensifying the increase of N in the river water. However, analyzing the samples collected from sampling points before and after the first fish farm showed that the δ^15^N values were similar, indicating no considerable variation among the areas. This suggests a complex N-receiving and cycling environment with multiple point and non-point sources. Others have found varying δ^15^N responses from point source N inputs in rivers^46^, likely due to high background N loading. Even though the high velocity and discharge of water in the river do not provide the necessary time for nitrification and also limit the retention of nutrients in the ecosystem, it seems that the main reasons for N contamination are agricultural land use in upstream areas and effluents from the fish farm in downstream areas.

The river's water flow has fallen significantly as precipitation has declined in recent years. Consequently, during the lowest water discharge of the river, about 100 L/s, the current capacity of the fish farm leads to maximum N concentrations (maximum capacity about 10 mg/L). According to the estimated annual load equal to 33 tons of N per year from the fish farm, the total values for six months were estimated to be equal to 16.5 tons of N. These values were very close to the estimated value of 14.6 tons of N using the Islam (2005) model and should translate to changes in the biological communities of receiving waters. Varol and Balci (2020)^29^ showed the influence of rainbow trout farms on reduced water quality (including increased chemical oxygen demand and nitrogen concentrations) and altered epilithic algal community composition. This was consistent with the assessment of Tahar et al. (2018)^30^, who reported effects of rainbow trout farms on water quality, specifically NH4-N and DO. Varol and Balci (2020)^29^ pointed to the need for low conversion rates and use of high quality extruded feed in fish farming, as well as increasing dilutions and recycling processes to improve water chemistry associated with fisheries waste.

Water quality modelling can be a valuable tool for water management since it can simulate the potential response of the aquatic system to such increases in nutrient levels. The use of generally available models should be verified with data obtained from the river for which its use is being considered. As mentioned, the predicted data were verified with the nitrogen data measured in the river. At the end of the rearing period in the summer when the discharge is at its minimum, the predicted nitrogen values were very close to the measured values in the Zarin-Gol River, which showed the validity of the model. Also, by putting the environmental parameters measured into the LOADEST model, the predicted values (in tons) were similar to those from the Islam (2005) model.

The analysis of δ^13^C in periphyton from various sampling points revealed that its values were not proximate to the δ^13^C values in benthic organisms, implying that most benthos did not feed on periphyton on the river bed rocks, which requires further examination. In rivers, benthic organisms generally feed on periphyton when it is available in open-canopied areas^47^, such as the Zarrin-Gol River. While it is plausible that regular scouring of periphyton transpires in this system due to frequent flooding, leading to invertebrates feeding primarily on detritus and other food sources, we observed complete periphyton coverage in several river reaches during our sampling. This is consistent with the eutrophic status of the river and suggestive of pressure on the ecosystem in terms of agricultural pollution and aquaculture effluents along the river.

The δ^13^C values in the fish species were similar to those of aquatic insect larvae, especially Trichoptera, but substantially different from algal organisms, indicating that the fish fed less on benthic algae. A large difference in δ^13^C values is not to be expected between producer and consumer levels (with some exceptions)^25^, and variation could be due to the amount of materials and substrate type of river beds in different areas^48^. The lack of a clear entry of periphyton C into the food web could be due to its nutritional quality, but taxonomic analyses would be required to assess this more fully.

In our research area, it appears that the filter-feeding Simuliidae found only at the most downstream site (S5) exhibited elevated δ^13^C and δ^15^N values that may indicate consumption of waste from the trout farms as it is broken down into fine particulate organic matter. Despite their relative distance in dual-isotope space, Simuliidae is not a favored food item for the fishes in the study river. More data for Simuliidae from other sites would be required to establish whether this taxon is part of a mixed diet for fishes that channels fish farm waste into higher trophic levels. Generally, the increase in δ^15^N from each trophic level to the next, which we observed, was consistent with the expectations from the literature^24,48^. Any changes in δ^15^N generally indicate the position of the organisms in the food chain. However, baseline δ^15^N values vary from site to site due to source inputs and cycling^19^. Species that co-occur within sites can have their δ^15^N values compared directly because they have the same baseline, so we can conclude that *P. hircanica* has a higher trophic status than *N. pallasi* and *C. razii*.

Freshwater systems typically have four trophic levels^19,49^. Based on this expectation, *P. hircanica* belongs to the top trophic level (3 and even 4 by feeding on eggs and larvae of other fish). While the other two fish species showed lower trophic levels, *N. pallasi* is expected to have a carnivorous dietary regime, belonging to trophic level 4 due to consumption of insects and fishes^50^ and *C. razii* often belongs to the upper range of trophic level 3 and sometimes to trophic level 2, though less is known about its diet. The fluctuation of trophic levels of fish species and highly variable baseline isotope values often indicate the entry of high amounts of N into the ecosystem^51^. Since *C. razii's* diet includes a combination of aquatic insect larvae and periphyton, both of which likely vary isotopically from site to site in the Zarrin-Gol, it is not possible to determine the role of each of them in the fluctuation in trophic levels of this species. However, determining trophic levels and the dominant diet items of the Zarrin-Gol River fish species can help to better manage the ecosystem. Particularly, finding which species (invertebrates and fishes) tend to graze periphyton could help with top-down control of algal growth in this eutrophic system. Based on our model, any further increases in fish farm production will directly increase N concentrations in the river, exacerbating an already stressed ecosystem.

The isotopic composition of nitrogen can vary depending on the source. There was large spatio-temporal variation in organic carbon and nitrogen isotopic ratios in the POM pool, which were controlled by storms and land use^52^. A study by Wang et al.(2020)^33^ showed sediment organic matter is affected by aquaculture waste even at a distance of 1500 m from the cage fish farm. Fertig et al. (2009)^53^ proposed that non-mobile benthic organisms can be used as biomarkers to identify contaminant sources on small scales. Considering this, the high δ^15^N and especially δ^13^C values in Simuliidae larvae can be an indication of the entry of sources of nutrients and organic matter into the river. These Simuliidae larvae had δ^13^C values very similar to trout farm waste measured elsewhere^43,44^ and require further study.

Generally, it is necessary to study the dynamics of the food web in aquatic ecosystems to determine the amount of energy flow among different trophic levels, and also to determine the production capacity of ecosystems. Considering this issue, the most important anthropogenic factors affecting the river ecosystem are the pollution of the river by large quantities of organic and inorganic substances. Though the fish farm effluent was difficult to detect with stable isotopes, it can be summarized that the river is in a critical situation and cannot bear the further development of the fish farms and land use along the river, especially during periods of low water discharge. Reduced discharges are expected due to climate change and water resource development in the southern Caspian basin and in many developing countries as well^54^. Therefore, this issue should be considered in aquaculture development plans in the future, and any expansion of land uses, including fish farming, could seriously damage river ecosystems.

## Conclusion

Agricultural runoff and fish breeding effluents are important factors releasing nitrate and phosphate that enters fresh waters, including rivers, and affect aquatic organisms. This study is among the first to assess new methods that combine modeling and empirical data to evaluate the effects of fish farming. Together, this has established a first reference for further research on river isotopes in dryland region rivers that experience combined nonpoint and point sources of nutrients. We encourage additional tests to confirm and develop this framework. It should be noted that there were limitations within our research; for example, the low quality of food and lack of purification systems in the fish farms may not be indicative of conditions elsewhere. Furthermore, the lack of laboratory facilities in developing countries, including Iran, and the high cost of transporting samples for isotopic analysis, are factors that limited our ability to achieve larger sample sizes. The unstable amounts of water flow due to rainfall and floods, and fertilization of riverside rice fields complicate the assessment of how nutrients are transported and cycled in this river system. However, continuous monitoring of the physicochemical parameters of water could identify the optimal production capacity of farms and prevent additional unauthorized increases in fish production. Therefore, to overcome the above-mentioned limitations in future research, we suggest the following: (1) measure constituents at different stages of the fish farming effluent to better understand if improved purification could be achieved, (2) obtain more accurate non-point source data on water pollution in the basin, to understand the role of agriculture, industries, aquaculture and forestry in nutrient runoff, (3) use additional ecological tracers to isolate the effects of the fish farms on nutrient uptake in the food web.

## Data Availability

All data generated or analyzed during this study are included in this published article.

## Appendix 1. Coding mathematical model in STELLA™ software version 9.0.1

ind_weight_N(t) = ind_weight_N(t − dt) + (assimilation) * dt

INIT ind_weight_N = .027

INFLOWS:

Assimilation = ind_food*0.16*0.3*0.9*0.5

Ntotal(t) = Ntotal(t − dt) + (Naccumulation) * dt

INIT Ntotal = 0.17

INFLOWS:

Naccumulation = IF(harvest = 0)THEN(pond_number*(excreation))ELSE(pond_number*(excreation + total_fecal + N_losese_or_pert))

number(t) = number(t − dt) + (− mortality) * dt

INIT number = 47000

OUTFLOWS:

Mortality = IF(ind_weight_B < 200)THEN(0.01*number)ELSE(number)

N_extcreated(t) = N_extcreated(t − dt) + (excreation) * dt

INIT N_extcreated = .077*total_food

INFLOWS:

Excreation = total_food*0.3*.16*.85 − (faeces_product + total_food*0.3*0.16*0.85*0.25)

N_losese_or_pert(t) = N_losese_or_pert(t − dt) + (unconsumed) * dt

INIT N_losese_or_pert = 0

INFLOWS:

Unconsumed = total_food*0.3*0.16*0.15

total_fecal(t) = total_fecal(t − dt) + (faeces_product) * dt

INIT total_fecal = 0.26*ind_food

INFLOWS:

Faeces_product = total_food*.3*.16*.85*.1

biomass = number*ind_weight_B*.2

harvest = IF(ind_weight_B < 700)THEN(0)ELSE(mortality*ind_weight_B)

ind_food = ind_weight_B*.03

ind_weight_B = ind_weight_N/0.0285

Natural_current = 100

N_concentration = (Ntotal/Natural_current/1000) + 2.28

pond_number = 1

total_food = biomass*ind_food*1.2
